# 

**Published:** 1897-03-06

**Authors:** 


### EARLY EDITION.

**Notices of Births, Deaths, and Marriages** are inserted in this column at a charge of 2s. 6d. for an announcement not exceeding four lines.

### NOTICE TO CORRESPONDENTS.

All MS., Letters, Books for Review, and other matters intended for the Editor should be addressed The Editor, "The Hospital," The Hospital Building, 28 & 29, Southampton Street, Strand, W.C.
The Editor cannot undertake to return rejected MS., even when accompanied by stamped directed envelope.

**Notice to Advertisers and Subscribers.**

All Advertisements, Orders for Copies of The Hospital, and Business Communications should be addressed to **THE MANAGER, (Not to The Editor),** The Hospital, The Hospital Building, 28 & 29, Southampton Street, Strand, London, W.C.
The Cost of Subscription, post free, is as follows:—Per week, 2½d.; per quarter, 2s. 8d.; per half-year, 5s. 3d.; per annum, 10s. 6d. Foreign:—Per annum, 15s. 2d.; payable, in all cases, in advance.

**NOTICE.—Vol. XX. of "The Hospital" (April to September, 1896), in handsome cloth binding, is now on sale at the Office of this Journal, price 7s. 6d. Cases for binding the Numbers contained in this Volume, 1s. 6d. Index to Vol. XX., 2½d., post free.**

**The Monthly Part for March .s now ready. Price 6d., by post 8d.**

### SCRAPS AND GLEANINGS.

IT is proposed to establish a Hospital for Incurables at Dundee.

A BAZAAR in aid of the Children's Hospital, Temple Street, Dublin, is to be held in May.

DURING the year 1896 103 patients were received into the St. Luke's Convalescent Home, Exmouth.

THE income of the Dewsbury and District General Infirmary for the year 1896 amounted to £2,310, and the expenditure to £2,225.

THE £10,000 required having been raised, the Dundee General Infirmary has undertaken to erect and maintain a maternity hospital in that town.

At Barrington's Hospital, Limerick, 595 in and 3,473 out-patients were treated during 1896 as against 581 in and 2,702 out-patients in 1895.

THE Samaritan Hospital for Women at Nottingham has only 105 subscribers, a fact not very creditable to a town of over 200,000 inhabitants.

THE committee of the Aldershot Hospital are endeavouring to raise an endowment fund for that institution as a commemoration of the Queen's long reign.

DURING the year just ended the ambulances of the Metropolitan Asylums Board have removed or transferred from one hospital to another 44,374 people.

THE Governors of the Essex and Colchester Hospital, after considerable discussion, have decided to limit the "life" of an out-patient's letter to six weeks instead of three months as heretofore.

THE tenders for the new building to take the place of the General Infirmary at Bedford have been accepted, and the work is to be commenced forthwith. The cost is estimated at £33,700.

THE recent ball held in aid of the Macclesfield Infirmary has added nearly £100 to the funds of that institution. About £300 was raised at Swansea in the same way in aid of the Swansea General Hospital.

HIS Highness the Chhatrapati Maharajah of Kolhapur has permitted his name to be placed on the list of patrons of the Imperial fête, and to grant a present of local manufactures in aid of the Victoria Hospital for Children.

### The Student of Medicine.

### APPOINTMENTS.

Helen M. Greene, L.S.A.Lond., M.D.Brux., appointed to the Staff of the Provident Dispensary, Derby. S. Edwards Jones, L.R.C.P., L.R.C.S Lond., L.F.P.S.Glasg., appointed Medical Officer for the Bangor and Worthenbury District of the Ellesmere Union. C. A. Lees, M.R.C.S., L.R.C.P., appointed Medical Officer for the Somersham District of the St. Ives Union. S. C. Legge, M.R.C S.Eng., L.R.C.P.Lond., appointed Clinical Assistant to the Birmingham and Midland Hospital for Skin and Urinary Diseases. Lily Leney, L.R.C.P., L.R.C.S.Edin., M.D. Brux., L.M.Rotunda, appointed Clinical Assistant to the New Hospital for Women, and Assistant Resident Medical Officer to the Canning Town Medical Mission. A. D. Miller, M.R.C.S., L.R.C.P.Lond., L.D.S.I., appointed Dental Surgeon to the Birmingham General Dispensary. Dr. Moon, appointed Resident Medical Superintendent to the Omagh Asylum. C. Ogle, M.A., M.B Oxon., M.R.C P.Lond., appointed Assistant Physician to St. George's Hospital, London. F. G. Penrose, F.R.C.P.Lond., appointed Physician to St. George's Hospital. I. G. Sibbald, M.B., C.M.Edin., appointed Resident Medical Officer to the Northern Infirmary, Inverness. Dr. T. Smyth, appointed Medical Officer for the Milton Abbot District of the Tavistock Union. D. Stanley, M.D.Edin., M.R.C.P. Lond., appointed Physician for Out-patients, Queen's Hospital, Birmingham. J. A. Turner, M.B., D.P.H., Medical Officer of Health to the Combined Districts of Leicester and Rutland, appointed Medical Officer of Health to the Combined Districts of East Herts and Essex. T. T. Whipham, M.A., M.D.Oxon., appointed Consulting Physician to St. George's Hospital.

### EXAMINATION OF CANDIDATES FOR HER MAJESTY'S ARMY AND INDIAN MEDICAL SERVICES, FEBRUARY, 1896.

The following papers were set:—

Medicine (Dr. Allchin).

1. State the diagnosis you would have made of the following case, the nature and situation of the lesion, and the reasons for your opinion. How would you have treated the case, and what prognosis would you have formed: J. W., aged 34, a commercial traveller, was admitted to the hospital in consequence of being paralysed in the left side. His family history was unimportant. Four years ago he is said to have had "pleurisy and inflammation of the lungs," and two years ago contracted syphilis, for which he was under treatment 18 months. He has lived well and drank freely, chiefly whiskey. His present illness came on suddenly three and a half months before admission when at the seaside. In the afternoon, whilst lying down after dinner (having bathed in the sea for nearly two hours earlier in the day) he was seized with pain in the loins and front of the head. He had previously been quite well. After the pain had lasted about half an hour, the left side of his body and face twitched, but only for a few seconds. He was not unconscious. After a short time he was able to get up, and managed to go for a short walk with his wife, though he moved his leg with some difficulty. He returned to London next day, and for more than a week attempted to follow his employment, but he finally had to give it up owing to increasing loss of power to his left side, which became complete in about fifteen days from his first attack. During this period it was noticed that the patient was very drowsy. He is reported also to have been somewhat childish in manner for two months previous to admission, and kept his bed entirely. Since the day after his attack he had been under medical treatment, but the nature of this is unknown. The day after admission patient was found to be a well-nourished man, not anæmic, without œdema of face trunk or extremities, but with a diffuse copper-coloured erythematous rash on upper part of body. He complained of some frontal headache. His manner was hesitating and slow, but he answered all questions rationally, there was some wandering and mild delirium at night with attempts to get out of bed. The face is slightly drawn to the right, and the left side is smoother. Both eyes can be closed tightly and equally, there is no paralysis of the ocular muscles, nor of those of mastication. Pupils are equal and re-act equally, though sluggishly to light and to accommodation. The tongue is protruded very considerably to the left, but lies evenly in the mouth. He says there is a little numbness of the left side of the face as well as in the left arm and leg. In these limbs there is complete loss of power involving every segment of each, but sensation is in no degree impaired. The left knee jerk is exaggerated and distinct ankle clonus is elicited on the same side, the left plantar reflex being very sluggish, much more so than on right side. There is no rigidity or wasting of the limbs. The electrical reactions are normal. There is no loss of control over the bladder and rectum, but patient says he cannot pass his water without at the same time passing a motion. Optic discs hyperæmic, veins full and outline of disc on nasal side is indistinct. Pulse 80, regular, of good volume but very soft. Heart sounds indistinct and distant, but no murmur audible. Lungs normal. Nothing abnormal found in abdomen. Urine, sp. gr. 1030, acid, no albumen, no sugar. Temperature normal.

2. Describe the microsopic characters of normal blood, and state what modifications are met with in chlorosis, pernicious anæmia, splenic anæmia, leucocythæmia, and lymphadenoma.

3. What are the morbid conditions leading to the formation of dilated bronchi? By what signs and symptoms is bronchiectasis recognised?

4. Describe in detail the treatment (dietetic, medicinal, and general) of a case of chronic nephritis, in which there is considerable anasarca and albuminous urine.

St. Thomas's Hospital.—The following gentlemen have been selected as House Officers from Tuesday, March 2nd, 1897: House Physicians: A. W. Sikes (extension), J. P. Scatchard (extension), J. S. Fairbairn, and E. Stainer. House Surgeons: A. J. Martineau, F. H. Gervis, R. G. Strange, and G. E. O. Taylor. Assistant House Surgeons: W. H. J. Paterson, A. W. Tuke, L. Gilbert, and S. N. Babington. Obstetric House Physicians: Senior, A. L. Home; junior, J. B. Tombleson. Ophthalmic House Surgeons: Senior, P. S. Hichens; junior, E. Hopkinson. Clinical Assistants in the Special Department for Diseases of the Throat: C. E. Jones (extension) and L. W. Richards (second term). Clinical Assistants in the Special Department for Diseases of the Skin: A. H. P. Dawnay (extension) and R. Whittington. Clinical Assistants in the Special Department for Diseases of the Ear: H. J. Macevoy and W. G. Stone. Clinical Assistants in the Electrical Department: C. G. Seligmann (extension) and F. W. Binckes.

